# Robot-assisted simple prostatectomy versus open simple prostatectomy: a single-center comparison

**DOI:** 10.1007/s00345-020-03168-1

**Published:** 2020-03-28

**Authors:** R. Dotzauer, A. La Torre, A. Thomas, M. P. Brandt, K. Böhm, R. Mager, H. Borgmann, W. Jäger, M. Kurosch, T. Höfner, C. Ruckes, A. Haferkamp, I. Tsaur

**Affiliations:** 1 Department of Urology, University Medical Center, Johannes Gutenberg University, Mainz, Germany; 2 Interdisciplinary Centre for Clinical Trials (IZKS), University Medical Centre of the Johannes Gutenberg‐University Mainz, Mainz, Germany

**Keywords:** Adenoma enucleation, Minimal invasive simple prostatectomy, Outcome, Complications

## Abstract

**Purpose:**

Open simple prostatectomy (OSP) is a standard surgical technique for patients with benign prostatic hyperplasia with prostate size larger than 80 ml. As a minimally invasive approach, robot-assisted simple prostatectomy (RASP) emerged as a feasible surgical alternative. Currently, there are no definite recommendations for the standard use of RASP. Therefore, we aimed at investigating various clinical outcomes comparing RASP with OSP.

**Methods:**

In this retrospective single-center study, we evaluated clinical data from 103 RASP and 31 OSP patients. Both cohorts were compared regarding different clinical characteristics with and without propensity score matching. To detect independent predictive factors for clinical outcomes, multivariate logistic regression analysis was performed.

**Results:**

Robot-assisted simple prostatectomy patients demonstrated a lower estimated blood loss and need for postoperative blood transfusions as well as less postoperative complications. OSP had a shorter operative time (125 min vs. 182 min) longer hospital stay (11 days vs. 9 days) and longer time to catheter removal (8 days vs. 6 days). In the multivariate analysis, RASP was identified as an independent predictor for longer operative time, lower estimated blood loss, shorter length of hospital stay, shorter time to catheter removal, less postoperative complications and blood transfusions.

**Conclusion:**

Robot-assisted simple prostatectomy is a safe alternative to OSP with less perioperative and postoperative morbidity. Whether OSP (shorter operative time) or RASP (shorter length of hospital stay) has a more favorable economic impact depends on the particular conditions of different health care systems. Further prospective comparative research is warranted to define the value of RASP in the current surgical management of benign prostatic hyperplasia.

## Introduction

Besides endoscopic enucleation of the prostate, open simple prostatectomy (OSP) is the standard surgical treatment for men with moderate to severe lower urinary tract symptoms (LUTS) and a prostate size larger than 80 ml [1]. Although, OSP has been proven over the years to be an effective surgical approach increasing the maximum flow rate and reducing the International Prostate Symptom Score (IPSS), there has been ongoing research for less invasive treatment options because of OSP’s significant side effects like bleeding, requirement for blood transfusion and revision surgery [2, 3]. As a minimal invasive alternative to OSP, robot-assisted simple prostatectomy (RASP) was first described by Sotelo et al. in 2008 [4]. Since then, different transperitoneal and extraperitoneal approaches have been developed [5–7]. Efficacy and safety of RASP have been investigated in different non-comparative studies and a multinational analysis.[8, 9]. However, there is a paucity of research comparing RASP with OSP. Hoy et al. retrospectively reviewed 4 RASP patients and 28 OSP patients [10]. In a larger retrospective study, propensity score-matched cohorts of 59 OSP patients and 59 RASP patients were compared [11]. In the only prospective comparative trial available, Mourmouris et al. compared 15 OSP patients and 26 RASP patients in a non-randomized multi-center study [12]. In view of the gap of sufficient data, RASP has investigational status in the current guidelines of the European Association of Urology (EAU) [1]. Therefore, we aimed to investigate the association between OSP vs. RASP by analyzing clinical perioperative and postoperative outcome characteristics. As cost effectiveness has become more and more important, we also addressed variables with economic impact such as length of hospital stay and operative time.

## Methods

### Data collection

After receiving approval of the institutional review board (Number: 2018-13808), all retrospective data were analyzed according to the Declaration of Helsinki. Clinical data of 134 consecutive patients who had undergone open simple prostatectomy or robot-assisted simple prostatectomy at the University Medical Center Mainz between April 2011 and January 2018 were collected from patient charts. There was no specific assignment of patients to either surgical procedure. RASP was primarily offered to all patients unless they had a history of previous extensive abdominal surgery or anesthesiologic contraindications against Trendelenburg position. However, due to overbooking of the robotic system by uroonologic procedures resulting in an extended waiting list for benign conditions, some patients decided to undergo OSP to avoid waiting. OSP was performed in transcapsular (*n* = 28) or transvesical (*n* = 3; in cases of concomitant bladder stones to alleviate their retrieval) technique as described by Millin or Freyer, respectively [13, 14]. OSPs were performed by five different surgeons with a case load of at least 50 open prostatic procedures. RASPs were performed by five different surgeons with a case load of at least 50 robot-assisted prostatic procedures. Low-molecular-weight heparins were commenced in all patients 6 h after surgery. If anticoagulants were used, they were preoperatively paused except for aspirin. RASP was performed with a transperitoneal transvesical approach as described by Thüroff et al. [7]. After having placed the patient in Trendelenburg position and established the pneumoperitoneum, the bladder is opened longitudinally from a transperitoneal approach. After identification of the ureteric orifices, the mucosa is incised circumferentially over the bladder neck and the adenoma is enucleated towards the apex of the prostate. After having re-approximated the urethral stump to the bladder neck, the bladder is closed with a running V-lock suture. Patients were admitted 1–3 days prior to surgery and discharged the day after catheter removal at the earliest. Catheter was removed in the absence of hematuria and after patency of the bladder, suture was tested by cystogram usually performed 5–7 after surgery. According to the clinic’s policy, routine follow-up was performed by practicing urologists who usually refer patients to our center. Nevertheless, some patients were followed up by our department in case of any complications or for individual patients’ requests. We included clinical patient characteristics as well as variables with potential economic impact (length of hospital stay, operative time) into the database. For clinical characteristics, we focused on preoperative (ASA classification system [15], Charlson comorbidity index [16], prostate size [ml] measured by transrectal ultrasound, preoperative urinary retention, preoperative post void residual urine [ml]), intraoperative (estimated blood loss [ml], intraoperative blood transfusions [yes vs. no], operative time [ml]) and postoperative variables (time to catheter removal [days], complications within 90 days according to the Clavien–Dindo classification system [grade < 2 vs. ≥ 2] [17], postoperative blood transfusions [yes vs. no], readmission rate within 90 days [yes vs. no], revision surgery [yes vs. no] and postoperative urinary retention [yes vs. no; need for transurethral catheterization within 90 days postoperatively]).

### Statistical analysis

We presented means and standard deviations for continuous variables and frequencies and proportions for categorical variables. Chi-squared and Mann–Whitney *U* test were used for univariate comparison between OSP and RASP patients. Propensity scores were calculated by a logistic regression model with terms for BMI, Age, Charlson Comorbidity Index, ASA classification, prostate size and preoperative urinary retention in the model. Propensity scores were added as a covariate in ANCOVAs for continuous variables and in logistic regression models for binary variables. For statistically significant outcome variables, multivariate logistic and linear regression analysis (using log-variables) was performed to identify independent predictive variables. Significance level was set to *p* < 0.05. Statistical analysis was performed using IBM SPSS Statistics Version 20 (Armonk, NY: IBM Corp.) and SAS 9.4 (Cary, NC: SAS Institute Inc.).

## Results

Preoperative characteristics from 31 patients who underwent open simple prostatectomy and 103 patients who underwent robot-assisted simple prostatectomy are presented in Table 1. There was no significant difference between both cohorts regarding age, BMI, prostate size, rate of preoperative urinary retention and amount of post void residual urine. Regarding comorbidity status, ASA classification and Charlson comorbidity scores did not differ between OSP and RASP patients.

**Table 1:** Comparison of clinicopathological characteristics of patients, open (*n* = 31) and robot-assisted simple prostatectomy (*n* = 103)

Variable	OSP	RASP	*p* value
Age, years (mean ± SD)	72 ± 6.9	71 ± 7.3	0.640
BMI, (mean ± SD)	27.8 ± 4.7	27.3 ± 3.2	0.897
Prostate size, ml (mean ± SD)	119 ± 25	127 ± 32	0.132
Preoperative urinary retention n (%)	17 (55%)	55 (53%)	1.000
Preoperative post void residual urine, ml (mean ± SD)	180 ± 176	185 ± 183	0.884
Preoperative IPSS Score (mean ± SD)	17.0 ± 6.6	17.3 ± 7.4	0.794
Preoperative urinary flow, ml/s (mean ± SD)	16.4 ± 16.8	6.1 ± 3.8	0.885
ASA classification system *n* (%)			0.219
1	11 (36%)	60 (59%)	
2	13 (43%)	34 (33%)	
3	1 (3%)	0	
4	1 (3%)	0	
Charlson comorbidity index *n* (%)			0.246
0	0	6 (6%)	
1	7 (22%)	17 (16%)	
2	8 (25%)	41 (40%)	
3	7 (22%)	16 (15%)	
4	4 (13%)	16 (15%)	
5	2 (6%)	5 (5%)	
6	2 (6%)	2 (2%)	
7	1 (3%)	0	

A shorter operative (125 min vs. 182 min) but a longer hospitalization time (11 days vs. 9 days) as well as time to catheter removal (8 days vs. 6 days) was observed for OSP than for RASP patients. While intraoperative transfusion rates were comparable, estimated blood loss was significantly higher for OSP than for RASP (682 ml vs. 248 ml). A higher rate of  postoperative complications (≥  2 Clavien–Dindo classification system 45% vs. 23%; significant for univariate testing without propensity score-matching) as well as that of postoperative blood transfusions (29% vs. 8% was demonstrated for OSP patients. There was no significant difference between the cohorts regarding revision surgery (12% vs. 10%), readmission rate (9.7% vs. 12.6%), postoperative incontinence (20% vs. 9%) and postoperative urinary retention (0 vs. 5%). Prostate cancer was diagnosed postoperatively in one patient in the OSP cohort and in ten patients in the RASP cohort. In all four revisions of the OSP cohort, bladder neck incision was performed; whereas in the RASP cohort, two patients underwent transurethral coagulation for macrohematuria, one patient underwent transurethral resection of the prostate for residual adenoma, three patients underwent surgery for incisional hernia and two patients underwent bladder neck incision. In the OSP cohort, one readmission was each necessary due to macrohematuria, urolithiasis, urinary tract infection and urinary retention. In the RASP cohort, five patients were readmitted due to macrohematuria, seven due to urinary tract infections and one due to urinary retention. The results are presented in Table 2.

**Table 2:** Comparing intra- and postoperative outcome variables in patients with open simple prostatectomy and robot-assisted simple prostatectomy

Variable	OSP	RASP	*p* value^1^	*p* value^2^
Operative time, min (mean ± SD)	**125 ± 53**	**182 ± 45**	**0.001**	**0.0006**
Length of hospital stay, days (mean ± SD)	**11 ± 5.8**	**9 ± 4.5**	**0.001**	**0.0048**
Time to catheter removal, days (mean ± SD)	**8 ± 4.1**	**6 ± 3.1**	**0.001**	**0.0082**
Estimated blood loss, ml (mean ± SD)	**682 ± 905**	**248 ± 363**	**0.007**	**0.0078**
Perioperative blood transfusion *n* (%)	1 (3%)	3 (3%)	1.000	0.6135
Complications ≥ 2 Clavien–Dindo *n* (%)	**14 (45%)**	**24 (23%)**	**0.024**	0.1752
Postoperative blood transfusion *n* (%)	**9 (29%)**	**8 (8%)**	**0.004**	**0.0090**
Revision surgery *n* (%)	4 (12%)	10 (10%)	0.738	0.4000
Postoperative urinary retention within 90 days *n* (%)	0	5 (5%)	0.589	0.9792
Readmission rate *n* (%)	3 (9.7%)	13 (12.6%)	1.000	0.8960
Postoperative urinary flow, ml/s (mean ± 95% CI)	14 (− 15 to 43)	18 (9–28)	0.747	0.4493

*p* values are presented (1 univariate comparison; 2 propensity score adjusted analysis). Statistically significant results are marked in bold

In multivariate regression analysis, RASP was identified as an independent predictor for longer operative time (coefficient 0.181; *p* < 0.001), shorter length of hospital stay (coefficient − 0.065, *p* = 0.029), shorter time to catheter removal (coefficient − 0.076; *p* = 0.020) and lower estimated blood loss (coefficient − 0.347; *p* = 0.001). Moreover, BMI was identified as an independent predictor for an increase in operative time (coefficient 0.007; *p* = 0.033). Furthermore, higher ASA scores predicted longer hospital stays (coefficient 0.069; *p* = 0.002). The results are presented in Table 3. OSP was identified as an independent predictor for postoperative blood transfusions (odds ratio 4.459; 95% confidence [1.258, 15.800]; *p* = 0.021) and postoperative complications (≥ 2 Clavien–Dindo classification system; odds ratio 2.662; 95% confidence interval [1.065, 6.654]; *p* = 0.036). Moreover, age was identified as an independent predictor for the need of postoperative blood transfusions (odds ratio 1.133; 95% confidence interval [1.013–1.268]; *p* = 0.029). Results are shown in Table 4.

**Table 3:** Multivariate regression analysis of pre- and intraoperative factors to predict operative time, length of hospital stay, time to catheter removal and estimated blood loss

Risk factors	End points							
	Operative time		Length of hospital stay		Time to catheter removal		Estimated blood loss	
	Coefficient	*p* value	Coefficient	*p* value	Coefficient	*p* value	Coefficient	*p* value
Surgical technique								
OSP	(Ref.)		(Ref.)		(Ref.)		(Ref.)	**0.001**
RASP	**0.181**	**< 0.001**	**− 0.065**	**0.029**	**− 0.076**	**0.020**	**− 0.347**	
Preoperative urinary retention								
No	1.00 (Ref.)		1.00 (Ref.)		1.00 (Ref.)		1.00 (Ref.)	
Yes	-0.021	0.324	− 0.018	0.460	− 0.033	0.229	− 0.025	0.772
Charlson comorbidity index	0.000	0.974	− 0.013	0.255	− 0.003	0.800	− 0.040	0.286
ASA classification system	− 0.014	0.484	**0.069**	**0.002**	0.016	0.523	0.047	0.534
Prostate size	0.000	0.324	− 0.001	0.190	0.000	0.511	0.001	0.618
BMI	**0.007**	**0.033**	− 0.003	0.451	0.003	0.467	0.003	0.780
Age	0.000	0.950	0.003	0.115	0.003	0.243	0.010	0.263

Statistically significant results are marked in bold

*n* = 130 cases

**Table 4:** Multivariate logistic regression analysis of pre- and intraoperative factors to predict postoperative blood transfusion and complications grade ≥ 2 according to Clavien–Dindo

Risk factors	Cases	End points			
	*n* = 130	Postoperative blood transfusion		Complications ≥ 2 Clavien–Dindo	
	*n* (%)	OR (95% CI)	*p* value	OR (95% CI)	*p* value
Surgical technique					
RASP	101 (77.7%)	1.00 (Ref.)		1.00 (Ref.)	**0.036**
OSP	29 (22.3%)	**4.459 (1.258–15.800)**	**0.021**	**2.662 (1.065–6.654)**	
Preoperative urinary retention					
No	61 (46.9%)	1.00 (Ref.)		1.00 (Ref.)	0.176
Yes	69 (53.1%)	3.059 (0.841–11.128)	0.090	1.772 (0.773–4.063)	
Charlson comorbidity index					
1–3	77 (59.2%)	1.00 (Ref.)		1.00 (Ref.)	0.374
≥ 4	53 (40.8%)	0.747 (0.174–3.213)	0.696	0.630 (0.228–1.742)	
ASA classification system					
1–2	81 (62.3%)	1.00 (Ref.)		1.00 (Ref.)	
≥ 3	49 (37.6%)	1.511 (0.371–6.152)	0.564	2.410 (0.887–6.545)	0.084
Prostate size	130	0.997 (0.977–1.018)	0.795	1.003 (0.989–1.017)	0.709
BMI	130	1.075 (0.909–1.271)	0.397	0.944 (0.840–1.061)	0.334
Age	130	**1.133 (1.013–1.268)**	**0.029**	1.014 (0.950–1.082)	0.679

Statistically significant results are marked in bold

## Discussion

We analyzed 134 patients treated at our institution comparing 31 open simple prostatectomies with 103 robot-assisted simple prostatectomies with the focus on their perioperative and postoperative characteristics as well as variables with economic impact. OSP was associated with a higher estimated blood loss and a higher rate of postoperative blood transfusion as well as postoperative complications grade ≥ 2 according to Clavien–Dindo. RASP was accompanied by a longer operative time, shorter hospital stay and time to catheter removal. An increase in BMI correlated with a longer operative time, higher ASA classification scoring with a prolonged hospital stay and higher age with a greater need for postoperative blood transfusions.

In line with our findings, Hoy et al. reported in a retrospective multi-center study of a shorter operative time, longer hospital stay and higher estimated blood loss for OSP patients. Notably, the reported cohort demonstrated a larger prostate size (OSP 239 ml vs. RASP 180 ml) than patients in our study (OSP 119 ml vs. RASP 127 ml). In contrast to our findings, no difference regarding 90-day complications and rate of postoperative blood transfusions between OSP and RASP patients has been reported [10]. Similarly, Sorokin and collaborators showed a shorter hospital stay, longer operative time and lower estimated blood loss for RASP patients in their propensity score-matched comparison but no significant difference of complications or rate of blood transfusions [11]. In line with existing studies, Mourmouris and co-workers reported of a longer operative time, shorter hospital stay and lower estimated blood loss for RASP patients. In this prospective comparative trial, a higher rate of grade ≥ 2 Clavien–Dindo complications in the OSP group could be observed [12]. Interestingly, the number of postoperative blood transfusions in our OSP cohort is higher than the one described in the literature (6.8–13%) [3, 18, 19]. This observation might be attributable to the limited size of our OSP group (*n* = 31) as well as varying patient characteristics and/or transfusion policy among institutions. Nevertheless, OSP was identified in our multivariate analyses as an independent predictor for estimated blood loss and postoperative blood transfusions (Table 5).

**Table 5:** Literature review of comparative studies of RASP vs. EP, OSP vs. EP and OSP vs. RASP

Author	Study design	Variables	OSP (n)	RASP (n)	EP	*p* value
Umari et al. JURO 2017 [20]	Retrospective, single center	Number of patients		81	81 (HoLEP)	
		Mean size of prostate (ml)		130	130	0.6
		Postoperative urinary flow rate (Qmax ml/s)		23	20	0.7
		Postoperative residual urine volume (ml)		0	0	0.4
		Transfusion rate (%)		1.2	0	0.5
		Complications % (Clavien ≤ 2)		21	20	0.7
Kuntz et al. Eur Urol 2007 [18]	Randomized prospective, 5-year follow-up	Number of patients	32		42 (HoLEP)	
		Mean size of prostate (ml)	113		114	0.6
		Postoperative urinary flow rate (ml/s)	24		24	0.97
		Postoperative residual urine volume (ml)	5		11	0.25
Sorokin et al. J Endourol 2017 [11]	Retrospective, single center	Number of patients	103	64		
		Mean size of prostate (ml)	144	136		0.396
		Postoperative urinary flow rate (Qmax ml/s)	20	22		0.36
		Postoperative residual urine volume (ml)	48	3.5		0.007
		Transfusion rate (%)	6.8	3.4		0.679
		Complications n (Clavien 3–5)	6	2		0.272
Current series	Retrospective, single center	Number of patients	31	103		
		Mean size of prostate (ml)	119	127		0.132
		Postoperative urinary flow rate (Qmean ml/s)	14	18		0.4493
		Transfusion rate (%)	29	8		0.0090
		Complications % (Clavien ≥ 2)	45	23		0.1752

Regarding surgical approach of RASP, several techniques have been proposed. Hoy et al. performed a transperitoneal approach and accessed prostatic tissue via capsulotomy distal to the bladder neck [10]. In the prospective multi-center trial, Mourmouris et al. reported also a transperitoneal approach. For enucleation, a vertical incision of the bladder and prostatic capsule was performed [12]. In contrast, we performed a transvesical approach to the adenoma without dropping the bladder off the pelvic wall in a transperitoneal approach, thus avoiding capsulotomy. Comparing clinical outcomes of the different studies, it should be considered that different open and robot-assisted techniques may have diverse risk profiles for perioperative or postoperative complications.

In the multivariate analysis, we identified a higher BMI as a predictor for a longer operative time. Similarly, Violette et al. described an association of a higher BMI with a longer operative time in 392 patients undergoing robot-assisted radical prostatectomy for prostate cancer [21]. Our findings that ASA scores predict for a length of hospital stay go in line with those of Carey and co-authors analyzing data of 1156 women undergoing gynecologic surgery [22]. In contrast to our findings of the age predicting for postoperative blood transfusions, Cassinelli et al. found no difference in transfusion rates in 166 patients undergoing surgery for spinal stenosis [23]. Regarding effectiveness and safety of procedures, our results demonstrated similar rates of revision surgery or postoperative urinary retention for RASP and OSP patients. In line with these findings, Shah et al. found RASP a safe alternative to OSP with less complications and similar effectiveness by reviewing 16 non-comparative and one comparative study [9].

Regarding various treatment options for patients with large prostate glands, transurethral enucleation techniques should be taken into account as valid alternatives recommended for the treatment of large prostates by the guidelines of the EAU. In a meta-analysis of seven randomized controlled trials comparing EP (plasmakinetic enucleation, bipolar enucleation or holmium laser enucleation of the prostate) to OSP, efficacy and safety of both approaches were comparable. In particular, there was no significant difference between both treatments regarding maximum flow rate, post void residual volume or IPSS. EP had a shorter catheterization time and hospital stay as well as a lower rate of postoperative blood transfusion. Interestingly, the rate of other complications was similar in both approaches [24].

Regarding economics, cost effectiveness is an important issue in robotic surgery and its costs have been a critically discussed issue that has to be set in relationship to its benefits compared to open surgery [25]. Interestingly, an online survey of 600 urologists figured out that decision-making in the management of benign prostatic hyperplasia (transurethral vs. open vs. robotic techniques) is not based on economic aspects but on safety, efficacy, own experience and availability [26]. For the robust economic comparison of RASP and OSP, further characteristics such as acquisition cost of the robot and its maintenance, stuff and instrument expenditures as well as reimbursement issues like compensation of the use of the robot should be investigated in further trials. Whether OSP with a shorter operative time or RASP with a shorter length of hospital stay potentially augmenting the annual case load of the department however higher expenses is associated with a better economic efficiency depends on the particular conditions of different health care systems as well as reimbursement policies.

Another aspect meriting evaluation is CO2 production during robotic procedures as the environmental impact of different surgical techniques should be taken into account. With regard to CO_2_ production and energy consumption, Woods et al. investigated 150 procedures (laparotomy, conventional laparoscopy and robot-assisted laparoscopy) demonstrating a consumption of 40.3 kg CO_2_/patient for robot-assisted surgery. This was 38% higher than CO_2_ production caused by conventional laparoscopy and 77% higher than that of open surgery [27].

Our study has several limitations. Due to its retrospective design, OSP and RASP may have undergone different peri- and postoperative management as data from a period of seven years was collected. Moreover, differences in the individual surgeon’s experience and intraoperative strategies could have affected our results. Furthermore, the majority of the OSPs were performed retropubically, whereas a transperitoneal transvesical approach was chosen for RASP. These anatomically different approaches could affect the kind or the number of complications. Another flaw is the lack of information about postoperative pain and analgesics request. However, our data reflect a real-world scenario of the routine health care of patients with benign prostatic hyperplasia. Although assessed in a non-randomized fashion, important patient characteristics did not differ between our both cohorts.

To the best of our knowledge, our study is comparing the largest number of RASP patients with OSP so far. Despite its limitations, this study underlines RASP as a safe and effective treatment method in comparison to OSP which should be offered to patients if EP is not available and robotic technique adopted. Another aspect is that long-term effects concerning urethral microtrauma and risk of consecutive urethral stricture are intuitively more likely to be a sequela of EP (rate of urethral strictures 1.8–2.2%) than of RASP (rate of urethral strictures 0.6%) which needs to take care of and be assessed in a prospective trial [8, 28].

## Conclusion

Robot-assisted simple prostatectomy is a safe alternative to OSP. It is associated with a lower estimated blood loss and need for postoperative blood transfusions as well as fewer postoperative complications. Furthermore, a longer operative time but shorter hospital stay for RASP could be demonstrated. For a better understanding of the economic value further characteristics such as acquisition cost of the robot and its maintenance, expenditures on stuff and instrument have to be illuminated. Moreover, safety and efficacy of RASP should be investigated in prospective randomized trials to rank RASP within the current treatment strategies of benign prostatic hyperplasia.
